# Centrifugal Partition Chromatography Is a Powerful Tool for the Isolation of Antibiofilm Quantum Carbon Dots Synthesized by Hydrothermal Treatment of Avocado Peels

**DOI:** 10.3390/molecules30071525

**Published:** 2025-03-29

**Authors:** Nandis Fiallos, Sergio Acuña, Diana Correa-Otero, Matías Venegas-Toloza, Tatiana Beldarrain, Josefina Burgos, Francisca Fuentes, Francisco Bustamante, Girlenne Christiansen, Vanesa Roa, Eduardo Schott, Julio Alarcón-Enos, Edgar Pastene-Navarrete

**Affiliations:** 1 Department of Basic Sciences, Faculty of Sciences, Universidad del Bío-Bío, Avenida Andrés Bello 720, Chillan 3800708, Chile; mahelymaravilla@gmail.com (N.F.); dianacorrea0114@gmail.com (D.C.-O.); mavenegas2017@udec.cl (M.V.-T.); josefina.burgos.f@gmail.com (J.B.); ffuentes2017@udec.cl (F.F.); francisco.bustamante2301@alumnos.ubiobio.cl (F.B.); gchristiansen@ubiobio.cl (G.C.); jualarcon@ubiobio.cl (J.A.-E.); 2 Facultad de Ciencias de la Salud y los Alimentos, University of Bío-Bío, Chillán 4050231, Chile; sacuna@ubiobio.cl (S.A.); tatybeldarrain@gmail.com (T.B.); 3 Departamento de Química Inorgánica, Facultad de Química y Farmacia, Centro de Energía UC, Centro de Investigación en Nanotecnología y Materiales Avanzados CIEN-UC, Pontificia Universidad Católica de Chile, Avenida Vicuña Mackenna 4860, Santiago 7510000, Chile; vnroa@uc.cl (V.R.); edschott@uc.cl (E.S.)

**Keywords:** centrifugal partition chromatography, carbon quantum dots, antimicrobials, biofilms, avocado

## Abstract

Carbon quantum dots (CQD) are an emergent nanomaterial with unique optical and biological properties. However, the purification of CQD is one of the bottlenecks that makes it difficult to scale for application in different areas. In this work, we explore for the first time the potential of centrifugal partition chromatography (CPC) as an alternative preparative technology to achieve the purification of CQD at the gram scale. The hydrothermal method was used to synthesize CQD from avocado peels. After 6 h at 250 °C, a complex mix of strong blue-fluorescent CQDs were obtained and submitted to CPC fractionation without pretreatment. The best results were obtained with the solvent system *n*-hexane–ethyl acetate–methanol–water (1:2:1:2, *v*/*v*/*v*/*v*), in an elution-extrusion protocol. Nine fractions were obtained and were characterized by UV-VIS spectrophotometry, Fourier transform infrared (F-TIR), and field emission scanning electron microscopy (FESEM), confirming the presence of CQD of different sizes. CPC fractionations indicate that a polarity-based separation mechanism can be used to purify CQD. Interestingly, four fractions showed antibacterial and anti-biofilm effects on *Pseudomonas putida* and *Listeria monocytogenes*. Therefore, CPC allows for better refining of this type of nanomaterial, and in combination with other techniques, it would serve to obtain CQD of higher purity, facilitating the physicochemical and bioactivity characterization of these particles. CPC would also allow the use of waste, such as avocado peels, to obtain new materials.

## 1. Introduction

The use of waste from the food industry has become one of the most promising research lines in green chemistry, seeking to reduce environmental pollution and recover bioactive molecules [1,2]. In addition, this trend has promoted the introduction of modern and environmentally friendly extraction technologies [3]. Among food wastes, peels and seeds of the avocado industry (*Persea americana* mill.) are a low-cost biomass susceptible to being exploited. Avocado consumption has increased in recent years, with this fruit being recognized for its high health benefits and bioactive content [4]. The adaptation of this fruit to tropical and subtropical areas allows it to be cultivated in more than 60 countries worldwide [2]. Chile ranks eleventh among the main avocado producers, with an annual production of 169,031.26 tons [5]. On the other hand, the industry only uses pulp to produce puree, oils, and snacks, which leads to the generation of by-products, which represent 25% of the total fruit. Among this agro-waste, avocado peel represents 11–17% [6]. The proximate composition of avocado peel includes carbohydrates (62–73.3%), protein (4–8.3%), lipids (4.4–9.1%), and ashes (4–6.1%). Moreover, it has high phytochemical content, and its fiber is rich in cellulose (28%), hemicellulose (25%) and lignin (4%) [7]. This makes avocado peel waste an interesting resource for the recovery of bioactive compounds such as polyphenols and lignocellulosic material [5]. In recent years, the synthesis of new green nanomaterials has gained increasing interest. Thus, different sources of organic molecules have been identified between biomass and non-biomass to synthesize quantum carbon dots (CQDs) with different optical and biological properties [8,9]. In this context, there are few studies related to the synthesis of CQDs from avocado peels by means of hydrothermal reaction. Recently, using different temperatures, CQD with excellent biocompatibility, optical, and photocatalytic properties were synthesized from avocado wastes [10,11].

Regarding CQD synthesis, bottom-up approaches are the most used since they have the advantage of being suitable for mass production, economical, ecological, sustainable, functional, and low-cost [12,13,14]. In particular, the hydrothermal carbonization method is considered a competent method with the advantages of self-generated pressure and subcritical temperature (180–250 °C), being viable from the point of view of energy savings. One of the advantages of using agro-wastes as a carbon source is that they contain hydroxyl, amino, carboxyl, or thiol functional groups on their surface, which allows the self-passivation of the CQDs present in the biomass [14,15,16]. Moreover, compared to unmodified CQDs, the incorporation of heteroatoms into the carbon structure confers to CQDs enhanced antioxidant and antimicrobial properties [17]. However, the mechanism of action of CQD is not fully understood. The main reason for this paucity is related to the CQD purification processes and the inherent complexity of the phenomena that these nanomaterials seem to harbor. This critical technological gap is associated with the purification steps aimed at separating CQDs from other materials that are formed as by-products of carbonization [18]. Techniques used, such as dialysis and ultrafiltration, fail to separate and concentrate CQDs effectively. Hence, certain molecules, such as low molecular weight fluorophores and materials with a wide distribution of size and surface heterogeneity, always persist as impurities that decrease the quantum yield of CQDs [19,20]. Therefore, it is imperative to emphasize the purification strategy to obtain CQDs with a narrow size distribution and unique optoelectronic properties, which aim to relate the structure/composition to specific properties and applications. Purification methods for CQD have been extensively reviewed in the literature [19,21,22]. These serial protocols, with several steps, make the purification process cumbersome and slow [22,23]. Regarding this latter, chromatographic methods have been repositioned in this field; however, their operation is expensive, and scaling requires the acquisition of preparative systems whose cost is beyond the budget of many projects. Despite these limitations, column chromatography allows CQDs to be separated based on polarity, charge, and size [19,20,22,24,25]. On the other hand, liquid-liquid extraction purification has also been considered as a strategy for cleaning and concentrating CQD [20,26,27]. Notably, the possibility of using a liquid–liquid-based chromatographic strategy has not been explored so far. Since some chromatographic separations of CQD are based on polarity, it is possible that if there are significant differences in the values of the distribution coefficient (*K_D_*), they can be separated into a two-phase liquid-liquid system. Centrifugal partition chromatography (CPC) is a countercurrent separation (CCS) technique characterized by its great load capacity and short separation times [28,29]. In this work, we obtain CQD from avocado peels as a substrate, which were subjected to classic hydrothermal treatment to obtain impure CQD. We aim to explore CPC as a strategy to obtain large amounts of pure compounds, whose scaling is much cheaper than the use of chromatographic columns filled with solid supports. After purification, morphology and optical properties of avocado peel CQD fractionated by CPC were characterized. Importantly, CPC allows the separation of CQD-enriched fractions that display antioxidant, antimicrobial, and anti-biofilm activities against *Listeria monocytogenes* and *Pseudomonas putida*.

## 2. Results and Discussion

### 2.1. Hydrothermal Synthesis of Carbon Quantum Dots (CQD) from Avocado Peels

The use of food waste with low added value to obtain nanomaterials has become an emerging strategy for obtaining materials of higher value, green, biocompatible, and easy to synthesize. The results of chemical composition for fresh avocado peel (Table 1), show a high moisture content (71.6%), followed by crude fiber, carbohydrates, lipids, proteins, and ash [30]. This carbon-rich composition favors the hydrothermal carbonization process for the formation of CQDs. The presence of proteins facilitates nitrogen (N) doping on the surface of CQDs, improving their functional properties and various applications [11]. Furthermore, avocado peel concentrates polyphenols from different types proanthocyanidins being the main group [31]. These compounds allow the surface of the CQDs to be functionalized with hydroxyl groups, which provide antioxidant or pro-oxidant properties depending on the context. Herein, the hydrothermal reaction conditions are based on previous work [10]. In our case, the hydrothermal reaction was carried out for 6 h at 250 °C, which is enough time and temperature to achieve the quantum confinement that allows the formation of CQD. During hydrothermal synthesis, the dehydration of the constituents of the avocado peel initially occurs, which are subsequently broken down to form smaller organic compounds such as furfurals and weak acids. The latter compounds polymerize inside the hydrothermal reactor to give rise to larger molecules that constitute the core of the CQDs [32].

### 2.2. Fractionation by Preparative Centrifugal Partition Chromatography (CPC)

**Figure 1:**
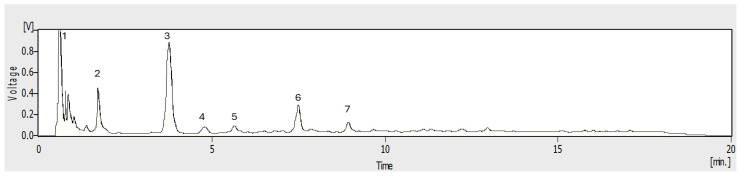
HPLC-UV chromatogram of total CQD from avocado peel at λ = 280 nm.

The hydrothermal reaction products of avocado peels were analyzed by C-18 HPLC-UV (Figure 1). The main peaks were (1) (t*_R_* = 1.02 min), (2) (t*_R_* = 2.21 min), (3) (t*_R_* = 4.25 min), (4) (t*_R_* = 4.92 min), (5) (t*_R_* = 6.51 min), (6) (t*_R_* = 7.58 min), and (7) (t*_R_* = 8.50 min). These peaks could putatively correspond to CQD, although it is highly possible that carbon core compounds (non-CQD) elute in the first two minutes [33]. On the other hand, the minor compounds observed from 10 to 22 min elute with higher percentages of acetonitrile and correspond to free fluorophores. Both types of compounds are considered impurities, and much of the effort invested in purifying CQDs is focused on their removal [19]. For this reason, this paper explores the potential of the CPC to accomplish this task. Thus, as a first objective, a solvent system that allows the best separation by CPC must be found. The solvent systems tested in CPC were taken from the Arizona series [34]. The Arizona family of solvents corresponds to a classification of biphasic liquid systems formed by alkane–ethyl acetate–methanol–water (Table S1) to which a letter of the alphabet from A-Z was assigned considering a decreasing order of polarity [34]. For cost reasons, in the present work, heptane was replaced by hexane. Initially, to establish the suitability of CPC for CQD fractionation, distribution coefficients calculation was conducted by the shake flask method. After preliminary visual experiments, we conclude that the characteristic fluorescence of CQD from avocado peel was evenly distributed in solvent systems A, C, G, K, and N. The C18 HPLC-UV analysis of upper and lower phases (Figure S1) of both the lower and upper phases allows us to rule out solvents N and A because the peaks are not distributed uniformly. For instance, with solvent system N, target peaks 3, 4, and 6 have *K_D_* values too low (below 0.25), which indicates that these compounds will predominantly be in the mobile phase in descending mode, so low retention and poor separation are assumed. On the contrary, for solvent system A, *K_D_* value of compound (6) exceeds 4; therefore, its elution will be too slow due to its higher retention in the stationary phase. After C-18 HPLC-UV fingerprint analysis of upper (organic) and lower (aqueous), the *K_D_* -values (Table 2) of target peaks at (3) (t*_R_* = 4.25 min) and (6) (t*_R_* = 7.58 min) were calculated using the peak area according to equation 2 (Section 3.5). The distribution of CQD shows that the solvent system K met better with the most important criterion of efficient CPC separation with *K_D_* in the range of 0.5–2.5 [28]. Most of the peaks of the CQD sample are in the correct range of 0.5–2.5, indicating a better balance between retention and elution (Figure S1). The *K_D_* values for the peaks at t*_R_* < 2 min were not calculated since these compounds probably are not CQD and have low retention in the C-18 column, preventing reliable integration. Together, all the results suggest that the descending mode with the lower phase of solvent system K as the mobile phase is the most appropriate for the separation of CQDs by CPC.

**Figure 2:**
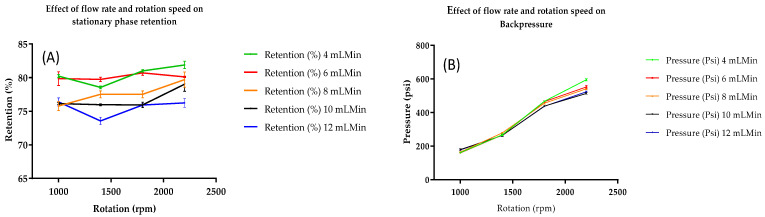
Effect of flow rate and rotation speed on stationary phase retention (**A**) and backpressure (**B**) using the solvent system K for CPC fractionation of CQD.

The effect of rotational speed and flow rate upon the stationary phase retention and backpressure were evaluated for solvent system K (Figure 2). In descending mode, rotational speed ranges from 1000 to 2200 rpm, whereas flow rate was studied from 4 to 12 mL/min. As expected, the higher the rotation speed, a slight improvement in stationary phase retention (0.6%) was observed. On the other hand, an increase of about 3.5 times in backpressure is observed. The effect of flow rate on stationary phase retention indicates that with flow rates of 4 and 6 mL/min, the retention remained above 80%, while flow rates from 8–12 mL/min generated a retention < 80%. Therefore, these latter flow rates generate greater stationary phase loss, leading to poor retention. The effect of flow rate was found to be more critical on stationary phase retention but was not significant on back pressure. Therefore, it is concluded that it is not worthwhile increasing the rotation beyond 1600 rpm and this can be set between 1600–1800 rpm without compromising stationary phase retention. In this study, the flow rate was set at 6 mL/min, while rotation speed was set at 1800 rpm. From these results, we use the selected solvent system K with the optimized conditions for semi-preparative fractionation of 500 mg of avocado CQD (10 mL injection loop). After equilibrium, the measured volume of stationary phase displacement was 46.5 mL. Considering that the experimental volume of the CPC-250 rotor was estimated to be 242 mL (see Section 3.5), the calculated stationary phase retention was 80.8%.

**Figure 3:**
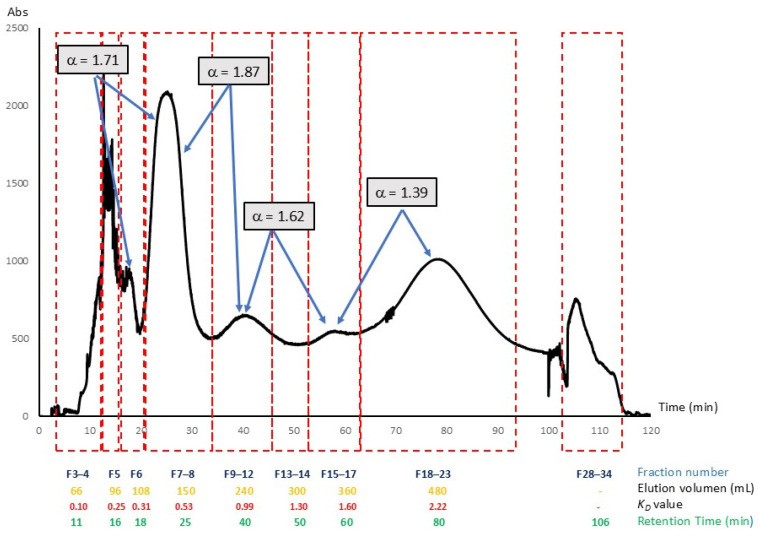
Chromatogram of CQD prepared by hydrothermal reaction of avocado peels. CQDs were fractionated in descending mode with the solvent system K. Flowrate: 6 mL/min, rotation speed 1800 rpm, and detection at λ = 280 nm. Stationary phase retention = 80.8%.

Nine fluorescent fractions were collected after one CPC procedure in descending mode. Using Equation (2) (Section 3.5), *K_D_* values of CPC-fractionated CQD were calculated from the elution volumes (Figure 3): F 3–4, *K_D_* = 0.10 (t*_R_* = 11 min), F5, 0.25 (t*_R_* = 16 min), F6, 0.31 (t*_R_* = 18 min), F7–8, 0.53 (t*_R_* = 25 min), F9–12, 0.99 (t*_R_* = 40 min), F15–17, 1.60 (t*_R_* = 60 min), and F18–23, 2.22 (t*_R_* = 80 min). Also, a fluorescent zone located in the valley between F13–14 (*K_D_* = 1.30) was collected. Target peaks (3, 4, and 6) defined in the HPLC analysis of shake flask experiments (Table 2) were found in fractions F7–8, F9–12, and F18–23, respectively. These CQDs show a slightly higher *K_D_* value compared with those of the shake flask experiments. Due to the stationary phase stripping, sample concentration in the injection, and design of the equipment partition cells, these discrepancies between the values of the shake-flask method and pulsed injection in CPC are not uncommon [35,36].

If purification of peaks with *K_D_* < 0.5 is required, the polarity of the solvent system must be reduced, or ascending mode elution should be attempted with a higher polarity solvent. However, such changes could impair the separation of the other compounds in the sample. Moreover, as mentioned above these compounds probably are non-fluorescent carbon core structures. Similarly, the C18-HPLC-UV analysis of F 28–33 (extrusion) shows a series of peaks over 10 min. While these compounds are not normally CQDs, they can be recovered in the extrusion step when CPC fractionation is performed.

**Figure 4:**
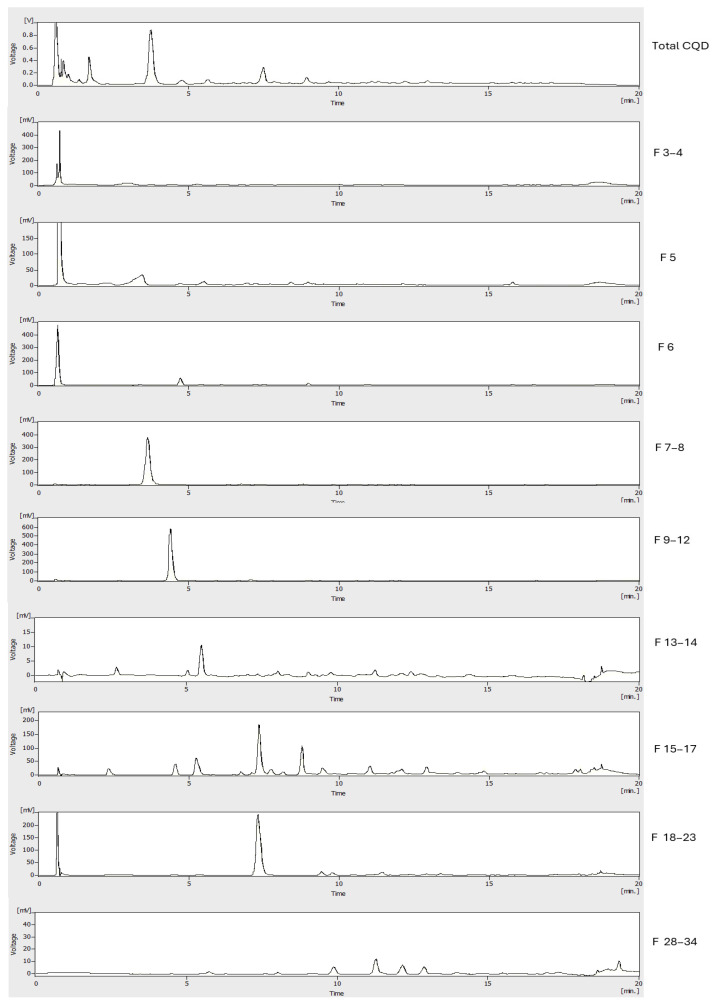
Analysis of pooled CQD fractions from CPC separation using solvent system K by C18 HPLC-UV at λ = 280 nm.

Interestingly, CPC fractions 7–8, 9–12, 13–14, and 19–23 have HPLC profiles (Figure 4) showing one peak. The separation factors (α) calculated with Equation (3) (Section 3.5) for the CQD pairs 6/7–8 (1.71), 7–8/9–12 (1.87), and 9–12/15–17 (1.62) were above the recommended value > 1.5 in the case of CPC separations [28]. The separation factor (α) calculated for compound pairs 15–17/18–23 (1.39) was lower than 1.5; therefore, less resolution and isolation efficiency will be expected. However, it must be pointed out that even for compounds with α values between peaks <1.5, advanced CPC formats such as sequential CPC (sCPC), multiple dual modes (MDM), or true moving bed chromatography (TMB) will allow achieving optimal separations [37]. Taken together, the results indicate that solvent system K allows the elution of the main CQD peaks in the sweet spot zone (*K_D_* = 0.5–5) within 100 min using approximately 3 column volumes. Descending mode in CPC using the lower phase of the solvent system K (ethyl acetate-MeOH-water 14-27-59 *v*/*v*), also has the advantage of using a hexane-free phase with a lower proportion of organic solvents. This facilitates the recycling of solvents, reducing the environmental impact. All the above, added to the predictability of CPC separations, allow for much more direct industrial scaling and transfer. Hitherto, the most used methodology to purify CQD is dialysis. Nevertheless, recent spectroscopic evidence such as nuclear magnetic resonance (NMR) and X-ray photoelectron spectroscopy (XPS) has raised criticisms over these classic CQD purification methods since their limited industrial application and the uncertainty regarding the quality of the separation from other low-weight molecular fluorophores that can be co-extracted from dialysis membranes [21,22]. As chromatography has repositioned itself in this field, its combination with dialysis membrane separation is already strongly recommended to improve the purity of CQDs.

As a way to corroborate whether the technique can be applied to a wider group of CQDs, we prepared phloroglucinol CQDs following the protocol of Yuan and coworkers [38]. The preparation of this type of CQD is very different, as it uses ethanol as a solvent and sulfuric acid as a catalyst. After 3 h at 200 *°C*, as expected, a mixture of CQD characterized by a strong yellow fluorescence and triangular shape was obtained [38]. Because these CQDs are prepared by a solvothermal method, one of their characteristics is their greater lipophilic character, so the solvent system L, formed by hexane-ethyl-MeOH-water acetate (2:3:2:3 *v*/*v*), allowed a better separation. As can be seen in Figure S2 (supplementary material), the fractionation of CQD from another carbon source is possible with CPC in ascending mode, even allowing the obtaining of high-purity fractions. This means that for the separation of CQD obtained from defined chemical components, CPC may have even greater potential and ease of application than in the separation of a more complex matrix such as avocado peels and other agri-food residues.

### 2.3. Characterization of Fractions with CQD Obtained by CPC:

#### 2.3.1. Analysis of UV-VIS Spectra for CQD Fractions Obtained by CPC

**Figure 5:**
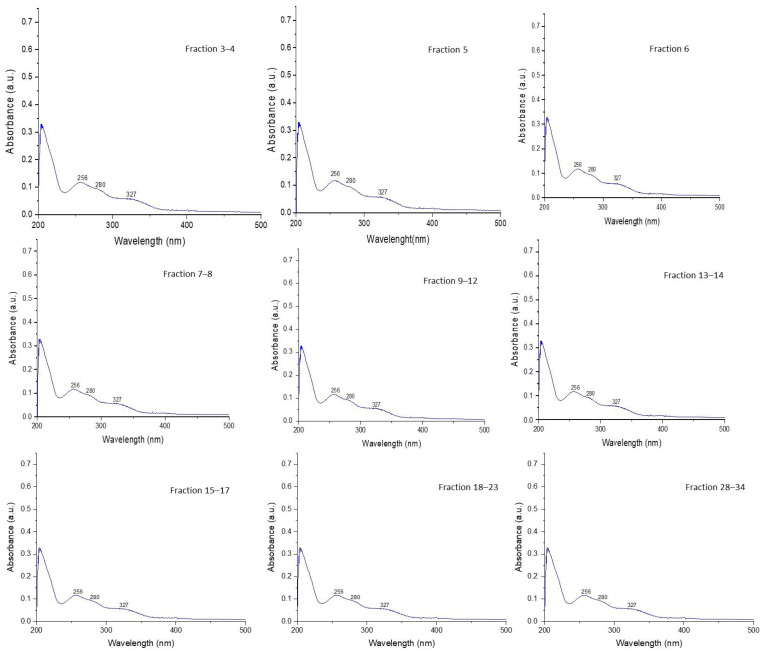
UV-vis absorption spectrum of avocado peel CQDs fractions purified by CPC.

Figure 5 represents the UV-Vis absorption spectra of the different avocado peel CQD fractionated by CPC. The absorption band located around 230–280 nm is attributed to the π–π* transition of the C–C/C=C bonds of the sp^2^ carbon core, while the absorption at 300–340 nm is generally due to the transition of n–π* of the C=O/C=N bonds formed in the hydrothermal synthesis process of CQDs. These features suggest that these nanoparticles have aromatic sp^2^ carbon domains in their structure, which will allow the absorption of UV radiation [39]. The wavelengths where these peaks are located remained unchanged, indicating that the electron orbitals and chemical structures are similar across fractions [40].

The UV-VIS spectra in Figure 5 were obtained in methanol; however, when examining the spectra obtained on-line during the CPC run (Figure S3), it was observed that some peaks have a different profile. The lower mobile phase used in CPC in the descending mode has an ethyl acetate-methanol-water composition of 14–27–59 *v*/*v*. It is very likely that this difference in polarity affects the spectrum of some peaks of CQDs obtained from avocado peels. The CPC fractions 3–4, 5, 6, and 28–34 have a similar profile and maximum absorption wavelengths located at 245, 277, and 326 nm. On the other hand, fractions 7–8 only present the maximum at 277 nm. Fractions 9–12 and 13–14 have maximums at 245 and 277 nm. Fractions 15–16 and 18–23 are the only ones that, in addition to the maximum at 277 nm, also have a band at 310 nm, which was not observed in the other fractions. This solvatochromic effect has been reported on several CQDs of different natures [41,42]. The effect accounts for a different surface distribution of the functional groups responsible for absorption in the UV-VIS range and how they behave in protic–aprotic solvents and at different pH [43]. This phenomenon also explains the change in fluorescence emissions observed in CQDs when they are dissolved in different solvents [44]. It has been reported that this change in solvent-dependent emission is explained by two emission centers in the CQD, the first being related to edge-state populations in the periphery of the center of the carbon-rich CQD with hybridization in sp^2^. The second element corresponds to the fluorescent groups on the outer surface. These features that explain the solvatochromic effect can be finely tuned by modifying the surface of the CQDs for being used as sensors [45].

#### 2.3.2. FE-SEM and XRD Analysis for CQD Fractions Obtained by CPC

**Figure 6:**
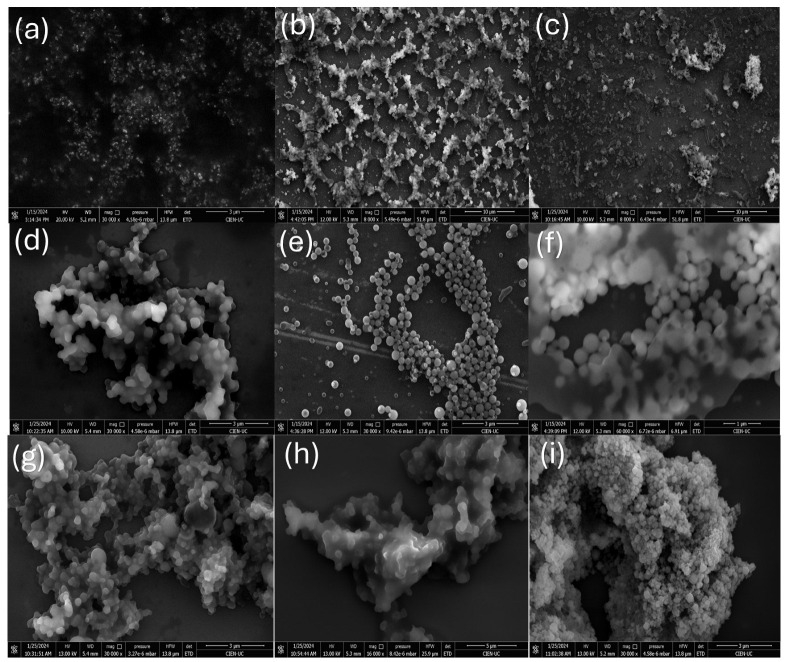
FE-SEM images of avocado peel CQD fractions obtained by CPC: (**a**) 3–4, (**b**) 5, (**c**) 6, (**d**) 7–8, (**e**) 9–12, (**f**) 13–14, (**g**) 15–17, (**h**) 18–25, and (**i**) 28–34. All images were obtained at 3 mm resolution, except (**f**,**h**), which were obtained at 5 mm and 1 mm, respectively.

**Figure 7:**
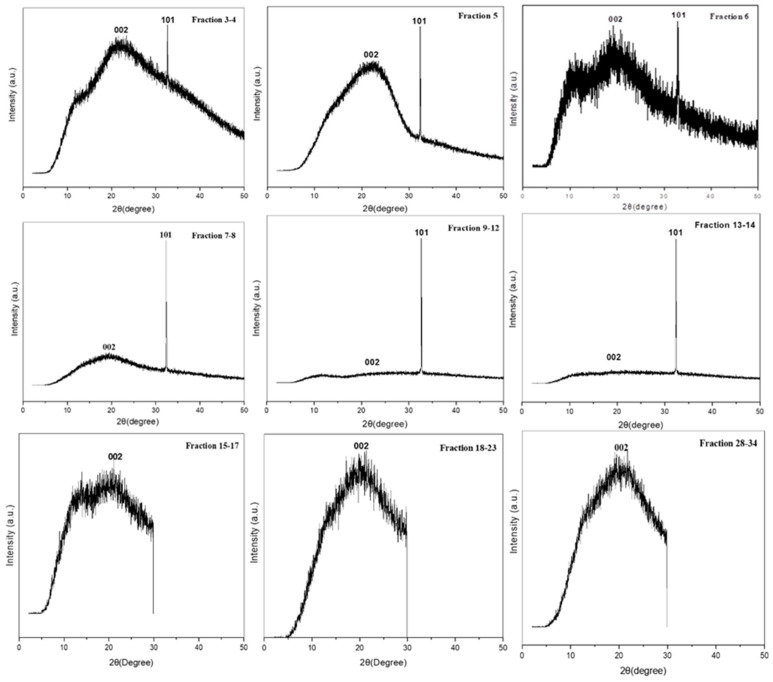
X-ray diffraction pattern of the different CPC fractions prepared from avocado peel CQDs.

The description of the morphology of the CPC-obtained fractions of avocado CQD is depicted in Figure 6. The Field emission scanning electron microscopy (FE-SEM) analysis highlights that the nanoparticles have a spherical shape with defined edges. In addition, a significant morphological transition between the different fractions is observed, which indicates that the purification performed by CPC was effective. Different degrees of aggregation of particles are also observed in some fractions. This agglomeration is governed primarily by π–π stacking interactions [46]. The amorphous/crystalline nature of the different fractions of avocado peel CQDs was investigated by X-ray diffraction (XRD), whose XRD patterns are shown in Figure 7. XRD analysis revealed that fractions 3–4, 5, 6, 15–17, 18–23, 28–34 have a wide and prominent diffraction peak (broad hump) centered in the plane (002) at 2Ө = 22.29°, 22.32°, 20.25°, 20.26°, and 20.17°, respectively. This peak is attributed to the interlayer packing of disordered carbon atoms in the hexagonal graphitic structure of the different fractions of CQDs, resulting in a turbo-static structure through interlayer packing (JCPDS No. 26-1076) [10,47]. In contrast, fractions 7–8, 9–12, and 13–14 exhibit a diffraction peak with weak intensity in the plane (002). In addition, the fractions 3–4, 5, 6, 7–8, 9–12, and 13–14 showed a narrow, sharp peak at 2Ө = 32°, indicating the presence of oxygen at the surface. In these CQD fractions carbon atoms are arranged in a rather disorderly fashion, along with an amorphous nature caused by the presence of more oxygen-containing clusters on the surface of CQDs [48]. However, fractions 15–17, 18–23, and 28–34 do not present evidence of oxygen on their surface.

#### 2.3.3. FT-IR Analysis for CQD Fractions Obtained by CPC

**Figure 8:**
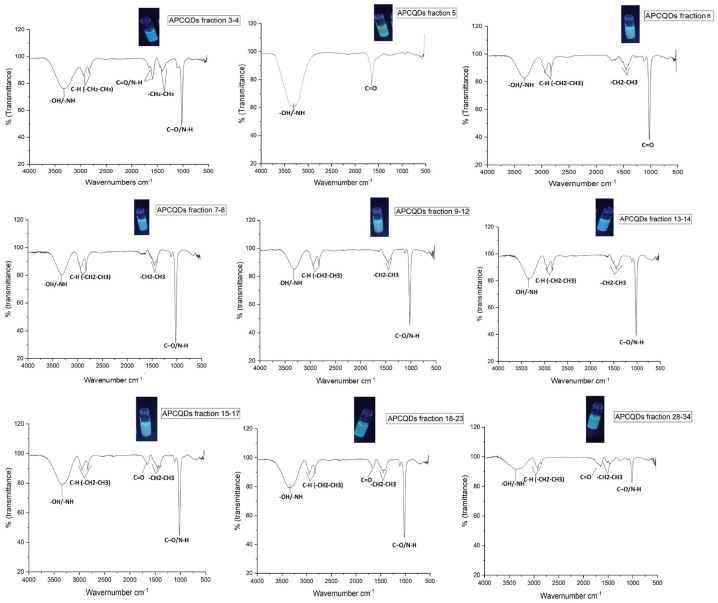
FTIR spectrum of avocado peel CQD fractions purified by CPC.

The carbon skeleton of the different CPC fractions from avocado peel CQDs is usually decorated with several surface functional groups. So, the FTIR spectra provided in Figure 8 show a slight displacement in a band with the absorption range 3200–3400 cm^−^^1^ which is characteristic of O–H and N–H stretch vibration mode. Peaks in the 2920–2090 cm*^−^*^1^ region are correlated with the C–H stretch vibration mode of CH_3_ and CH_2_, except for fraction 5, which lacks this band. For fractions 5, 15–17, 18–23, and 28–34, an absorption band was observed around 1635.64 cm^−1^, 1647.21 cm*^−^*^1^, 1641.42 cm*^−^*^1^, respectively, which is assigned to the carbonyl stretch mode (C=O) of a secondary amide, typical of all amines. This peak often appears in the range of 1680 to 1630 cm*^−^*^1^. In detail, for fractions 3–4, C=O was also observed at 1666.07 cm*^−^*^1^ with an accompanying peak around 1592.78 cm*^−^*^1^, this peak describes the simple N–H flexion bonds in the secondary amide plane [49]. In addition, the region between 1400 and 1500 cm*^−^*^1^ exhibited distinctive peaks associated with the C–H bending vibrations of the functional groups such as CH_2_ and CH_3_ in the different fractions except for fraction 5 [11]. The peaks centered at 1010 cm*^−^*^1^ and 1020 cm*^−^*^1^ in the different fractions were attributed to stretchings of the C–O ether or C–N groups [50]. This latter confirms the heterogeneity of surface functional groups that confer high solubility in water to the avocado peel CQDs fractions.

### 2.4. Antioxidant Activity

**Figure 9:**
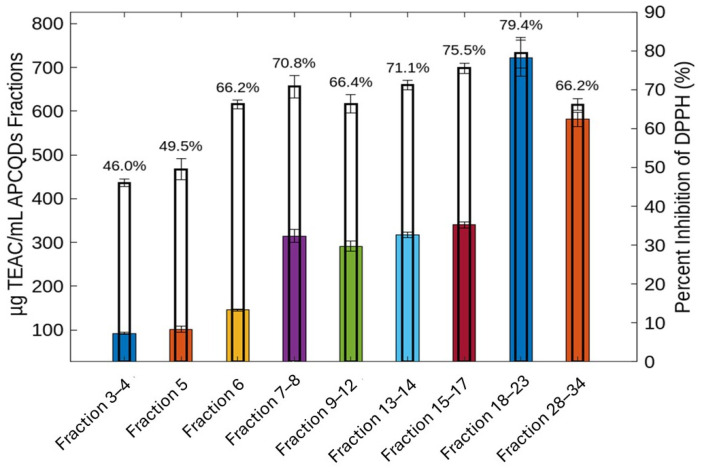
Antioxidant activity of CQD fractions (10 µg/mL) determined by the DPPH method. Different colors for bars represent the TEAC values for each CPC fraction.

Figure 9 shows the antioxidant activity of CPC-fractionated CQDs using the DPPH assay. Samples of the CPC fractions of CQD were prepared at a concentration of 10 µg/mL and compared. The DPPH scavenging activity of fractions 3–4 and 5 was significantly lower compared to the other fractions. On the other hand, fractions 7–8, 9–12, 13–14, and 28–34 showed an average antioxidant activity, which could be due to a lower or higher density of functional groups such as hydroxyls (-OH) and carboxyl (-COOH), which are essential for the transfer of electrons and hydrogen, respectively. Regarding the 15–17 and 18–23 fractions, they have a significantly higher performance in antioxidant capacity. Since spectroscopic analyses (UV-VIS and FT-IR) show similar characteristics for all CPC fractions, the difference in antioxidant capacity could be attributed to their higher degree of functionalization and chemical homogeneity. Several previous studies have reported the antioxidant effects of CQDs obtained from different matrices [51]. The commonly accepted mechanism of action of CQDs as antioxidants considers the reduction of DPPH. to DPPH-H by the transfer of hydrogen from the -COOH, -OH, and -NH_2_/-NH surface groups. Subsequently, the unpaired electrons in the CQD groups can be delocalized by resonance in the aromatic environment or by rearrangement of the chemical bonds of the same functional groups, managing to stabilize the structure [52,53]. It should be noted that this mechanism is general and cannot be extrapolated to all CQDs because it is very difficult to adjust the surface chemical characteristics that are essential for the antioxidant effect to manifest itself [54]. Indeed, it is still difficult to explain how the combination of surface chemical groups and the physicochemical environment determines the Janus-like behavior of CQDs as antioxidants and pro-oxidants (see Section 2.5) [55,56].

### 2.5. Antimicrobial Properties

#### 2.5.1. Antibacterial Properties of CPC CQD Fractions

We evaluated the antibacterial effect of the nine fractions of CQDs obtanied by CPC against two bacterial pathogenic strains, *L. monocytogenes* and *P. putida* (Table 3). *L. monocytogenes* is a Gram-positive facultative intracellular pathogen. Infection is frequent in individuals with lowered immunity, such as transplanted patients or elderly people [57]. The control of this bacterium in food is of special concern since outbreaks of listeriosis associated with the ingestion of ready-to-eat (RTE) products occur from time to time, which has led the authorities to establish permanent monitoring policies for this pathogen [58]. Within the control measures for *L. monocytogenes*, the development of nanoparticles loaded with copper, selenium, or zinc has become an attractive strategy for the development of active packaging that helps limit the development of pathogens [59]. On the other hand, *P. putida* is an environmental Gram-negative bacterium of low virulence. This strain used as a model in the investigation of compounds that inhibit the production of biofilm. Like *P. aeruginosa*, biofilm formation can be induced by antibiotics, which helps improve the tolerance of internal cells [60]. In order to investigate the effect of CQD from avocado peels, we performed an antimicrobial screening assay. The antibacterial activity was measured as the diameter of the inhibition zone (mm) and compared with the standard antibiotics azithromycin and ampicillin. The results revealed that CQDs showed greater effectiveness against *L. monocytogenes*, while its effect against *P. putida* was less significant. This could be attributed to the higher structural resistance of Gram-negative bacteria due to their outer membrane rich in lipopolysaccharides [61]. Fractions 28–34, 3–4, and 9–10 stand out for their activity, showing inhibition zones comparable to or greater than those of azithromycin against *L. monocytogenes*. The highest antibacterial activity against *L. monocytogenes* was observed in fractions 28–34, which is the most lyophilic fraction. However, CQDs are generally less effective than ampicillin against *L. monocytogenes*. The bactericidal effect of avocado peel CQDs can be exerted through various main pathways, including physical and mechanical damage to the bacterial membrane. This latter effect could be explained by CQDs intercalation into the bacterial membrane [62]. This interaction occurs when CQD binds to the phospholipid bilayer, which makes it rougher and shrinks, causing the rupture and discharge of its cellular components. The lysis of the bacterial cell wall caused by synthetic compounds and CQD has been previously attributed to the disruption of electrolyte balance, which ultimately leads to the death of the organism [63]. Other mechanisms include: inactivation induced by photothermal effects, direct or light-induced generation of reactive oxygen species (ROS) [64], as well as damage and fragmentation of DNA/RNA and proteins [65]. The core structure and surface functional groups of CQDs determine their functional and biological properties. Furthermore, the antibacterial efficacy of CQDs will depend on the structure, precursor, synthetic methods, functionalization, size, doping, shape, zeta potential, and surface charge effects [66].

The results of the minimum inhibitory concentration (MIC) of CQD fractions against pathogenic bacteria (Table 4) suggest that *P. putida* exhibits high resistance to all CQD fractions, with MIC values exceeding 500 µg/mL. As mentioned above, this result may be related to the fact that Gram-negative bacteria have an outer membrane composed of lipopolysaccharides, which act as a physical and chemical barrier, hindering the penetration of CQD. Another possible reason could be the contribution of efflux pumps and metabolic mechanisms to their resistance [63,67]. On the other hand, in our study, the antimicrobial effectiveness against Gram-positive bacteria *L. monocytogenes* was higher. The fractions with the highest antimicrobial activity are 28–34, 13–14, and 15–17, especially against *L. monocytogenes*. These fractions show significantly lower MIC values (33.61 ± 1.05 µg/mL, 83.49 ± 0.79 µg/mL, and 64.28 ± 1.83 µg/mL, respectively). Statistically significant differences (different letters) highlight that CQD fractions (such as fractions 28–34 and 15–17) are more effective against *L. monocytogenes*, which could be related to differences in the functionalization of their surface. These fractions possess specific chemical characteristics, such as a higher density of charged functional groups (amino, carboxyl) and a greater capacity to generate reactive oxygen species (ROS), which can destabilize bacterial membranes [54,68]. The lower activity of fractions 3–4, 5, and 6 suggests these lack optimal characteristics for bactericidal interactions. The ROS production could be explained since CQDs are good photosensitizers. These nanoparticles have high electron transfer capacity with large amounts of free electrons and holes. In addition, under ultraviolet or visible light, they lead to bacterial oxidative stress by inhibiting their respiration, replication, and subsequent cell apoptosis through the formation and accumulation of hydrogen peroxide (H_2_O_2_), hydroxyl anions (OH^−^), singlet oxygen (^1^ O_2_) and triplet oxygen (^3^ O_2_) [65,69]. It should be emphasized in any case that the mechanism by which CQDs generate ROS and how the passivation layer and its thickness affect this property is not entirely clear. Recent evidence suggests that along with polarity, size, and shape of the CQDs are strongly related to their bactericidal activity. Thus, those CQDs that have a Gaussian curvature ≠ 0 are more likely to match with bacteria such as *S aureus*. On the other hand, when the Gaussian curvature is approximately equal to 0, CQDs exert a negligible effect on bacteria such as *P. aeruginosa* [70].

#### 2.5.2. Anti-Biofilm Properties of CPC CQD Fractions

**Figure 10:**
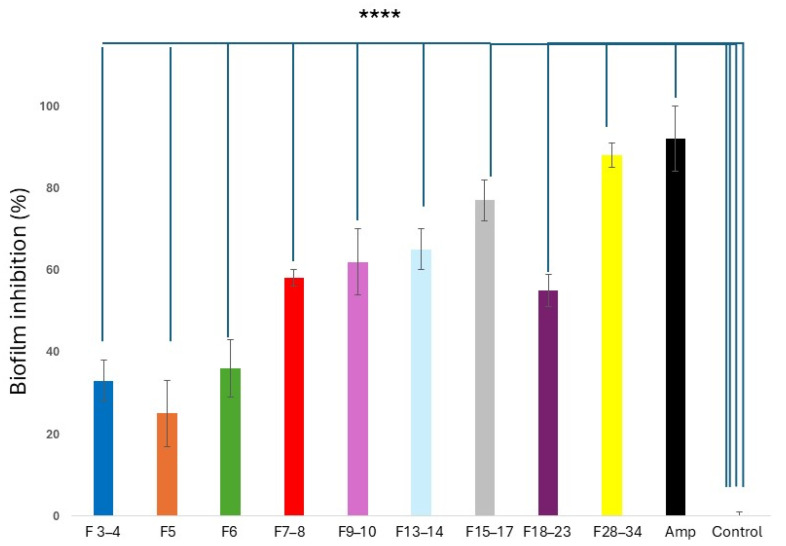
Anti-biofilm (crystal violet assay) activity of CPC fractionated avocado peel CQD at 500 μg/mL on *L. monocytogenes* 19115. The % of biofilm inhibition was evaluated after 12 h of incubation at 37 °C. Data are presented as mean ± SD. The asterisks indicate a significant difference from the control (untreated), with **** indicating *p* < 0.0001.

In Figure 10, the anti-biofilm effects of avocado peel CQD over *L. monocytogenes* are shown. As can be seen, all fractions promoted a significantly different inhibition (*p* < 0.0001) than that observed with the control of *L. monocytogenes* without treatment. Regarding ampicillin, only the F28–34 fraction showed a similar effect on the biofilm of *L. monocytogenes*. The anti-biofilm mechanism of action of CQDs is related not only to their role as nano-enzymes in the generation of free radicals but also to more specific molecular events [71,72]. For example, the transcriptomic study of *Porphyromonas gingivalis* exposed to tinidazole carbon dots allows the anti-biofilm effect to be related to the inhibition of genes such as *Fim A*, *Rgp A*, *Rgp B*, and *KGP*, all linked to the expression of proteins necessary to build the biofilm [73]. One of the advantages of CQDs is their hyperpermeability, which facilitates their penetration into the superhydrophobic structure of the biofilm to reach their molecular targets [74]. One aspect that must be taken care of with this type of nanomaterial is its safety. Many publications refer to their safety, but in those cases where CQDs display antimicrobial effects, their effects on the normal microbiota should also be analyzed. In this regard, ε-poly-l-lysine CDs have been reported to severely alter the probiotic properties of *Lactobacillus rhamnosus* strains, promoting dysbiosis and intestinal inflammation [75].

## 3. Materials and Methods

### 3.1. Chemicals

Acetonitrile, ethanol, methanol, and acetic acid (99.9%) were HPLC grade and acquired from Merck (Darmstadt, Germany). For CPC, n-hexane (Hex), ethyl acetate (EtOAc), and hydrochloric acid (HCl) were analytical grade and purchased from Merck (Darmstadt, Germany). Sulfuric acid (95–88%), gallic acid (97.5%), phloroglucinol (99.0%), 6-hydroxy-2,5,7,8-tetramethylchroman-2-carboxylic acid (Trolox), and 1,1-diphenyl-2-picrylhydrazyl (DPPH) were purchased from Sigma-Aldrich (St. Louis, MO, USA).

### 3.2. Proximate Analysis of Avocado Peel

The chemical composition of the avocado peel was carried out triplicate through the analysis of moisture, fat, ash, carbohydrates, protein, and crude fiber, and following standard AOAC methods [76]. The total polyphenol content was determined by the methodology of Singleton and Rossi with minor modifications [77]. Total phenol content was expressed as milligrams of gallic acid equivalent (GAE) per 100 g of wet avocado peel (mg GAE/100 g WM).

### 3.3. Hydrothermal Synthesis of Carbon Quantum Dots (CQD) from Avocado Peels and Phloroglucinol

Freeze-dried avocado peels were obtained from avocado fruits (*Persea americana* Mill. var Hass) purchased at a local market in Chillán, Chile. Batches of 2 g peels were transferred to a Teflon-lined autoclave (50 mL). Afterward, 40 mL of deionized H_2_O was poured inside; the autoclave was tightly sealed, placed in an oven, and heated at 250 °C for 6 h. After cooling at room temperature, the reactor was opened, and the obtained product was filtered using filter paper. The solution obtained was then centrifuged at 10,000 rpm for 30 min and finally filtered using a polyvinylidene difluoride (PVDF) membrane of 0.22 µm. This clear solution was freeze-dried for 48 h, and the yield of the carbon dots was calculated from the initial amount of the starting material. The dark brown CQD was stored in the fridge at 4 °C until CPC fractionation and characterization. In order to confirm the versatility of CPC for fractionation, phloroglucinol CQDs were synthesized according to previous work [38]. These CQD were neutralized with saturated NaHCO_3_ solution, filtered, and concentrated under vacuum. Fractionation was conducted by CPC without further purification procedures, except filtration through 0.22 µm PVDF membrane.

### 3.4. Characterization of Carbon Quantum Dot Fractions Derived from Avocado Peels

The powder X-ray diffraction (PXRD) characterization for the identification of the different fractions of avocado peel CQDs was carried out in a D2 PHASER Bruker with Mo Kα1 radiation. Fourier transform infrared spectroscopy (FT-IR) spectra were performed with an attenuated reflectance (ATR) accessory on an IR Spirit equipment, SHIMADZU (Kyoto, Japan). The surface morphology of the different fractions was analyzed by field emission scanning electron microscopy (FE-SEM-actual Thermofisher, model Quanta 250 FEG, Waltham, MA, USA), The core-level binding energy was adjusted according to the C 1 s binding energy of 284.8 eV corresponding to adventitious carbon.

### 3.5. CPC Separation Procedure

For the fractionation of CQD from avocado peels, a centrifugal chromatograph CPC 250 Gilson (Villiers le Bel, France) with 800 twin cells and 250 mL total volume was used. After subtracting extra-column volumes (tubing and channels), the experimental column volume available for liquid-liquid chromatography was = 242 mL. The system has four-way switching valves for either operating in descending or ascending modes. The separation process, detection, and collection of the fractions were controlled with a PLC-2050 system (Gilson, France). CQD separations were conducted using different ratios of the two-phase solvent system composed of *n-*hexane-ethyl acetate–methanol–water (HEMWat). The solvent systems were prepared using the shake-flask method allowing the phases to equilibrate overnight. For all experiments, the CPC rotor was operated in descending mode and initially filled with 300 mL of the upper stationary phase at 30 mL/min and 500 rpm rotation. To optimize operation conditions, the lower mobile phases were pumped at a flow rate between 4–12 mL/min (CPC) and rotational speed from 1000 to 2200 rpm. The back pressure was recorded during this step, and the percent of stationary phase retention was calculated after equilibrium. Once setting conditions were established, 500 mg CQD sample was dissolved in a 10 mL 1:1 mixture of upper and lower phases and injected into the CPC system in descending mode. In the case of phloroglucinol CQD, the solvent system *n*-hexane-ethyl acetate–methanol–water (system L, 2:3:2:3 *v*/*v*) was used in ascending mode. Chromatograms were obtained with scan 200–600, 280, 350, and 430 nm wavelengths. The volume of collected fractions was 25 mL tubes. Tubes were gathered according to on-line UV spectra and the fluorescence emission under a UV lamp (Spectroline, Model CM-10A, Melville, NY, USA) of 365 nm.

*K_D_* calculations using shake-flask experiments: The distribution coefficients (*K_D_*) were calculated according to Ito and coworkers [28], with slight modifications. In brief, four 200 µL aliquots of CQD were evaporated under a vacuum in a Centrivap. To the dried residues, 1 mL of the different biphasic solvent systems were added and thoroughly equilibrated by vortexing for 1 min. Phase separation was carried out by centrifugation at 12,000 rpm for 5 min. Fifty microliters of upper and lower phases were diluted with 200 mL methanol and analyzed by HPLC with the method described in Section 3.6. Four different solvent systems were tested (Table 2). Based on the ratio of HPLC peaks area in lower and upper phases, the *K_D_* values were calculated using Equation (1):

(1) $$K=\frac{Concentration\;of\;compound\;in\;stationary\;phase}{\;Concentration\;of\;compound\;in\;mobile\;phase}$$

*K_D_* calculation from CPC [78]: Using the optimal separation conditions described above, pulse injections were performed with a 5 mL sample loop. The sample was prepared by dissolving 80 mg of CQD in 5 mL of a 1:1 mixture of upper and lower phases.

(2) $$K_{D}=\frac{F\;tR-\left(1-SF\right)VC}{SF\;VC}$$

where *F* is the flow rate, *t_R_* is the retention time of the target CQD, *SF* is the retention of the stationary phase, and *VC* is the total column volume.

Separation factor (*α*) calculation [79]: The calculation of the separation factor (*α*) for neighboring peaks is defined as the ratio of the distribution coefficients (3):

(3) $$\alpha =\frac{K_{D}1}{K_{D}2}$$

where *K_D_*1 corresponds to the solute with the highest distribution coefficient (*K_D_*1 greater *K_D_*2).

### 3.6. High-Performance Liquid Chromatography (HPLC-UV)

The analysis of the CQD fractions was carried out by the HPLC YL9111S system (Young Lin^®^, Anyang-si, Republic of Korea). All separations were performed using a Kromasil KR100 column 100 Å—3.5 μm-C18, 4.6 mm × 150 mm (Eka Chemicals AB, Bohus, Sweden). As a mobile phase, 0.5 % acetic acid (A) and 100% ACN (B) in gradient elution mode were applied. The gradient program used was as follows: 0–2 min, 2% B; 2–22 min, 2–20% B; 22–24 min, 20–30% B; 24–26 min, 30–60% B; 26–32 min, 60–2% B; 32–37 min, 2% B. The wavelengths in the UV/Vis detector were set at 280 and 350 nm. The mobile phase flowrate was 0.7 mL/min, the injection volume was 20 μL, and the column temperature was maintained at 35 °C.

### 3.7. Antioxidant Activity: DPPH Radical Inhibition

The CPC fractions with the avocado peel CQD were tested using a 1,1-diphenyl-2-picryl hydrazyl (DPPH) assay according to Min et al. [49]. In 96-well plates, aliquots of 20 μL of properly diluted samples of CQD, control (Trolox), and blanks (methanol) were combined with 280 μL of DPPH and mixed. This mixture was agitated for 1 min and kept in the dark for 20 min. After that, the absorbance of the microplates was measured at 517 nm using an EPOCH Elisa reader (BioTek Instruments, Charlotte, VT, USA). The data were analyzed using the Gen5 software package version 1.11. The percentage of inhibition of the DPPH radical was calculated using the following Equation (4):

Free radical scavenging activity (%) = (Abs sample − Abs control)/(Abs control) × 100(4)

where Abs sample is the absorbance of the sample containing the CQD, and the Abs control is the absorbance of the DPPH reagent dissolved in methanol. The results were expressed in μg Trolox equivalent antioxidant capacity (TEAC)/mL sample.

### 3.8. Microbial Culture and Maintenance

The strains used in this study were *Listeria monocytogenes* ATCC 19115 and *Pseudomonas putida* ATCC 4704. The strains were stored in 20% glycerol at −80 °C (Laboratory of Synthesis and Biotransformation of Natural Products). When used in the antibacterial assays, they were incubated under aerobic conditions in trypticase soy broth (TSA; BD^©^, Franklin Lakes, NJ, USA) at 37 °C for 24 h. In the diffusion test, *Listeria monocytogenes* was streaked onto PALCAM agar (Merck©) or Trypticase Soy agar (BD^©^) plus Yeast Extract (Merck^®^), whereas Mueller–Hinton Agar (Oxoid^®^, Waltham, MA, USA) was used as a culture medium for *Pseudomonas putida* and [80].

### 3.9. Screening Disk and Well-Diffusion Test Susceptibility

The antibacterial potential of CQD and CPC fractions was analyzed in terms of the zone of inhibition following the disc diffusion assay of the Kirby Bauer method [81]. Control antibiotic susceptibility disks of 10 μg ampicillin and 15 μg azithromycin were used. In this assay, 100 mm and 25 mL plates of MRS, PALCAM (Barcelona, Spain), and Müeller–Hinton (BD^©^) were used. Bacterial suspensions were prepared in sterile saline adjusted to McFarland 0.5 for the strains *L. monocytogenes* 19115 and *P. aeruginosa* ATCC 4704, and were spread using a sterile swab. Subsequently, discs impregnated with CQD were deposited onto agar plates. Pathogenic strains were cultured at 37 °C for 24 h in aerobic conditions [81]. The antibacterial activity of CQD was conducted in biological triplicate and expressed as the mean diameters (mm) of growth inhibition around the disks produced by the tested samples.

### 3.10. Minimum Inhibitory Concentration (MIC)

For minimum inhibitory concentration, 100 µL of each carbon dot concentration (500 µg/mL to 1.95 µg/L) was added to individual wells of a 96-well plate. The bacterial culture was adjusted to an optical density equivalent to 1 × 10^8^ CFU × mL^−1^ and then diluted to 1 × 10^6^ CFU × mL^−1^. Optical density values were confirmed with a bacterial count, all within optical density 0.5 ± 0.02. One hundred microliters of bacterial suspension were added to each well. A negative control included broth only was prepared. As a negative control, sterile water was used, and as a positive control, amoxicillin (10 µg/mL) was used. The microplate was incubated at 37 °C for 24 h. Bacterial growth was observed visually, and absorbance readings were at 600 nm using a microplate reader Varioskan LUX 1.00.38 (Thermo Fisher Scientific, Santiago, Chile) [82].

### 3.11. Biofilm Test on Biofilm Formation

The impact of CPC-obtained avocado peel CQD fractions on the ability of *L. monocytogenes* 19115 to form biofilm was investigated using 96-well plates with bacterial inoculum as previously described [83]. Stock solution of CQD was prepared at 5.000 µg/mL in sterile saline, while ampicillin was diluted to 1.000 µg/mL. The cells were incubated in BHI [84] with each CPC fraction of CQD diluted at 500 µg/mL final concentration at 37 °C for 12 h. Ampicillin was used at 100 µg/mL final concentration. After incubation, unbound cells were removed by aspiration and allowed to dry for 10 min. Then, the wells were rinsed 3 times with 200 µL of sterile PBS (Phosphate buffered saline, pH 7.4) and dried at 60 °C for 45 min. The biofilm biomass was stained with 200 µL of 0.1% crystal violet (CV, Merck^©^). After 10 min, CV was eliminated by aspiration, and the plates were air-dried for an additional 10 min. The excess CV was rinsed with 200 µL of PBS (X4) and allowed to dry for 10 min. Biofilm-bound CV was resolubilized with 200 µL of ethanol: acetone mixture (80:20). Biofilm inhibition was quantitated by measuring the absorbance at 590 nm using a microplate reader Varioskan LUX 1.00.38 (Thermo Fisher Scientific, Santiago, Chile) [85].

## 4. Statistical Analysis

All data were initially entered in Excel. Statistical analysis was performed in GraphPad Prism Software version 8.0.1. To compare every mean with a control, Dunnett’s post hoc test was performed after run ANOVA. Experiments were performed in triplicate and expressed as means ± SDs. The results were statistically significant when *p* < 0.05.

## 5. Conclusions

In this work, for the first time, the power of CPC in the separation of CQD obtained from avocado skin is evidenced. The separation was carried out with conventional two-phase systems (solvent system K), demonstrating that the mechanism based on the distribution coefficient allows its fractionation. Traditional dialysis and ultrafiltration processes fail to remove non-CQD fluorescent impurities, which is currently one of the bottlenecks in CQD purification. However, the use of classical methods in conjunction with chromatographic separations appears to be the best strategy from now on. In this sense, CPC has several advantages over systems that use columns with solid stationary phases, which makes it possible to scale it directly and even optimize the purification of CQD that are very similar to each other. CPC, as a purification tool, will not only help improve CQD yields but also will facilitate the physicochemical characterization of these materials and the elimination of free non-fluorescent material. To illustrate this in the present work, the antioxidants and antimicrobial activity against two pathogenic strains were confirmed. Given the green character and great versatility of CPC, it could be applied to any type of CQD in the future.

## Figures and Tables

**Table 1:** Chemical composition of avocado peel.

%	Mean ± SD
Ash	3.7 ± 0.59
Protein	7.9 ± 0.39
Moisture	71.6 ± 0.73
Fat	18.1 ± 0.63
Crude fiber	39.0 ± 0.02
Carbohydrates	3.1 ± 1.41
Polyphenols (g/100 g) *	3.3 ± 0.52

* Yield calculated on fresh weight basis of avocado peels.

**Table 2:** The calculated *K_D_* values for the major components of avocado peel CQD in different solvent systems.

Solvent Systems	Peak 3	Peak 4	Peak 6
A	1.60	1.63	4.19
C	1.25	1.38	2.88
G	1.06	1.22	2.67
K	0.48	1.05	1.92
N	0.20	0.22	0.29

*K_D_* values obtained from the analysis of peak compounds in upper and lower phases by C-18-HPLC-UV as described in Section 3.5.

**Table 3:** Results of antimicrobial susceptibility tests for CQD fractionated by CPC.

CQDs CPC Fractions	*P. putida* Inhibition Zone (Mean ± SD, mm)	*L. monocytogenes* Inhibition Zone (Mean ± SD, mm)
3–4	10.83 ± 0.40 ^bcd^	17.47 ± 0.78 ^de^
5	10.97 ± 0.21 ^bcd^	16.43 ± 0.14 ^bc^
6	11.13 ± 0.83 ^bcd^	15.73 ± 0.14 ^b^
7–8	11.70 ± 0.46 ^b^	17.33 ± 0.64 ^cd^
9–10	11.20 ± 0.36 ^cd^	17.07 ± 0.64 ^cd^
13–14	10.60 ± 0.26 ^bc^	15.97 ± 0.57 ^b^
15–17	9.50 ± 0.26 ^a^	17.37 ± 0.21 ^cde^
18–23	10.27 ± 0.78 ^ab^	18.50 ± 0.07 ^e^
28–34	10.87 ± 0.40 ^bcd^	19.96 ± 0.61 ^f^
Azithromycin	13.30 ± 0.14 ^b^	12.97 ± 1.34 ^a^
Ampicillin	-	23.27 ± 0.61 ^g^

Different lowercase letters in the same column indicate significant differences (*p* ≤ 0.05).

**Table 4:** Minimum inhibitory concentration (MIC) of CQD fractions obtained by CPC tested against pathogen bacteria.

CQDs CPC Fractions	*P. putida* mg/mL	*L. monocytogenes* mg/mL
3–4	>500	124.32 ± 0.95 ^f^
5	>500	125.48 ± 0.87 ^f^
6	>500	126.35 ± 0.40 ^f^
7–8	>500	88.46 ± 1.71 ^e^
9–10	>500	82.26 ± 0.95 ^d^
13–14	>500	83.49 ± 0.79 ^d^
15–17	>500	64.28 ± 1.83 ^b^
18–23	>500	72.85 ± 1.74 ^c^
28–34	>500	33.61 ± 1.05 ^a^
Amoxicillin	>500	1.22 ± 0.80 ^g^

Different lowercase letters in the same column indicate significant differences (*p* ≤ 0.05).

## Data Availability

The data presented in this study are contained within the article and in Supplementary Material.

## Supplementary Materials

The following supporting information can be downloaded at: https://www.mdpi.com/article/10.3390/molecules30071525/s1, Table S1: Arizona solvent systems; Figure S1: HPLC-UV trace of CQD from avocado peel in upper and lower phases of the solvent systems A, C, G, K and N. Figure S2: CPC chromatogram of CQD synthetized from phloroglucinol and fractionated using hexane-ethyl-MeOH-water acetate (solvent system L, 2:3:2:3 *v*/*v*); Figure S3: On-line UV-vis absorption spectrum of avocado peel CQDs fractions obtained during CPC separation with solvent system K.
