# Supplementary material for: Centrifugal Partition Chromatography Is a Powerful Tool for the Isolation of Antibiofilm Quantum Carbon Dots Synthesized by Hydrothermal Treatment of Avocado Peels
Source: Molecules. 2025 Mar 29;30(7):1525. doi: 10.3390/molecules30071525 (PMC11990913; doi:10.3390/molecules30071525)
Supplement: Supplementary file 1 [file molecules-30-01525-s001.zip › molecules-3482360-supplementary.pdf]

Supplementary Material

Table S1. Arizona solvent systems

Arizona System	*n*-hexane*	Ethyl acetate	Methanol	Water
A	0	1	0	1
B	1	19	1	19
C	1	9	1	9
D	1	6	1	6
E				
F	1	5	1	5
G	1	4	1	4
H	1	3	1	3
I				
J	2	5	2	5
K	1	3	1	3
L	2	3	2	3
M	5	6	5	6
N	1	1	1	1
O				
P	6	5	6	5
Q	3	2	3	2
R	2	1	2	1
S	5	2	5	2
T	3	1	3	1
U	4	1	4	1
V	5	1	5	1
W	6	1	6	1
X	9	1	9	1
Y	19	1	19	1
Z	1	0	1	0

**Heptane was replaced by n-hexane. Red letters and numbers indicate the systems used in the present study*

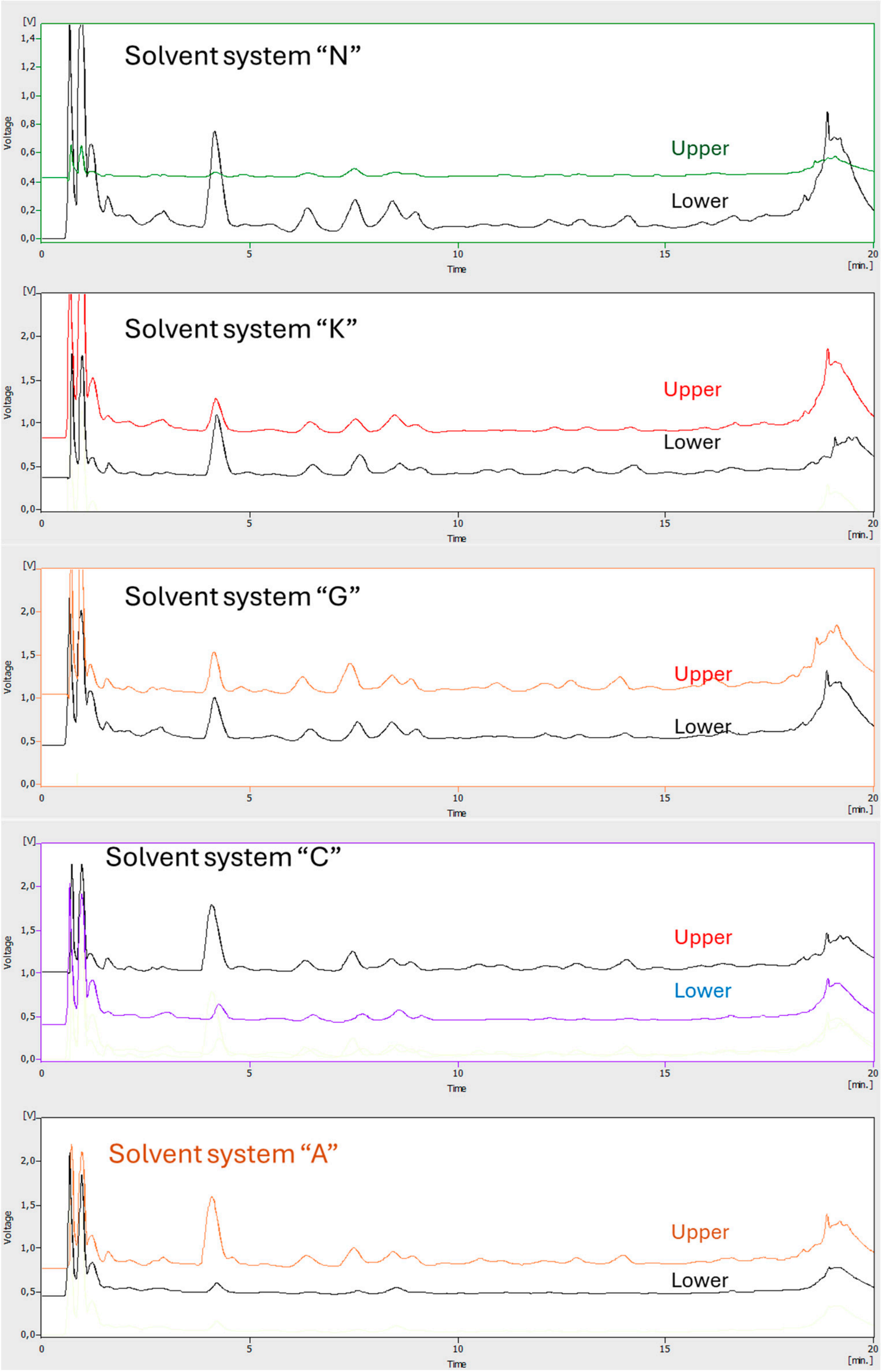

Figure S1: HPLC-UV trace of CQD from avocado peel in upper and lower phases of the solvent systems A, C, G, K and N. Chromatograms were recorded at 280 nm wavelength. Chromatograms were recorded at 280 nm wavelength.

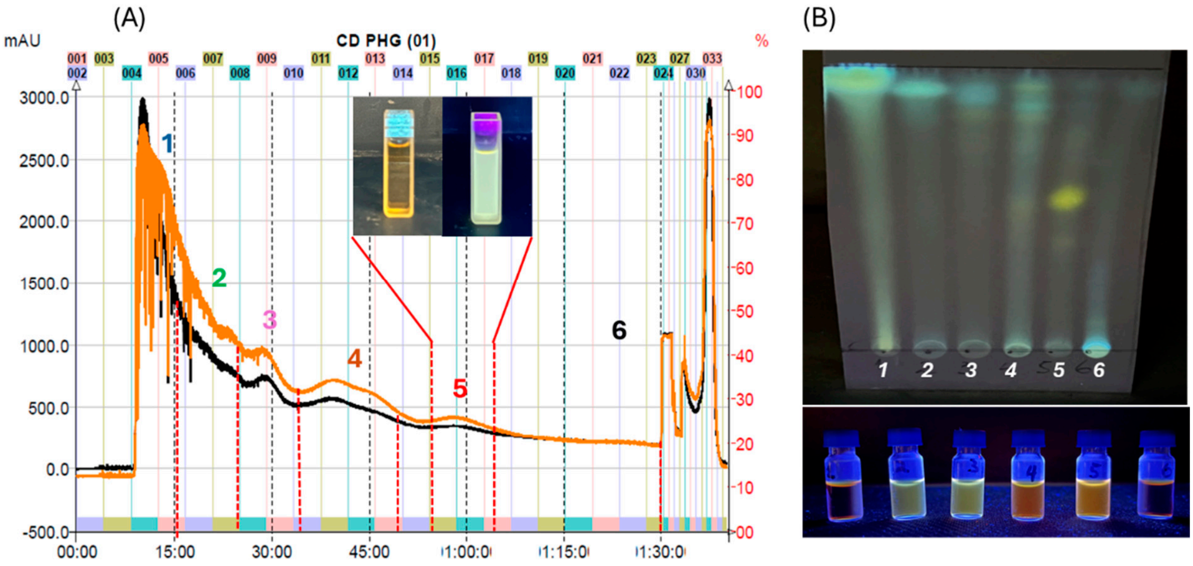

Figure S2. CPC Chromatogram of CQD synthetized from phloroglucinol and fractionated using hexane-ethyl acetate-MeOH-water (system L, 2:3:2:3 v/v) (A). TLC analysis of CPC fractions of CQD separated by CPC. Mobile phase for TLC was hexane/ethyl acetate/methanol/water (1.5-1.5-1.5-1 v/v). Lanes 1-5 correspond to the fractions collected after (B) CPC separation. The inset in (A) represent illustrative photographs of fraction 5 in daylight (left) and irradiated under UV 365 nm lamp (right).

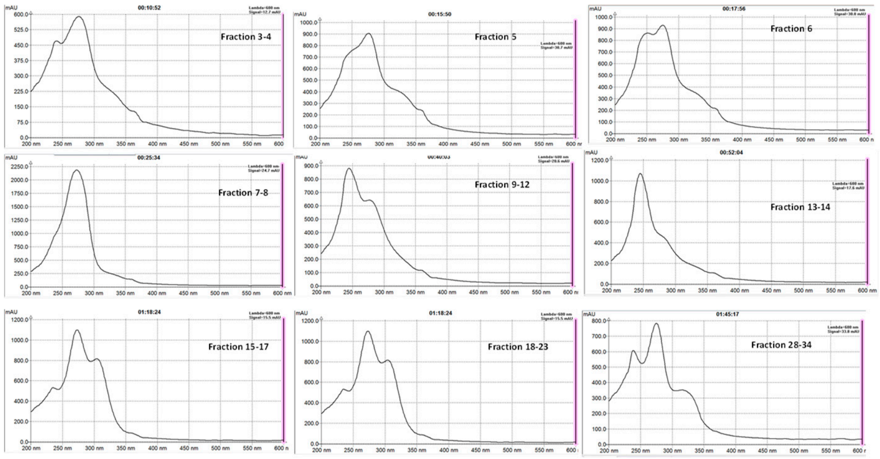

Figure S3. On-line UV-vis absorption spectrum of avocado peel CQDs fractions obtained during CPC separation with solvent system K.
